# Correction: A transcription factor network specifying inhibitory versus excitatory neurons in the dorsal spinal cord

**DOI:** 10.1242/dev.155986

**Published:** 2017-07-01

**Authors:** Mark D. Borromeo, David M. Meredith, Diogo S. Castro, Joshua C. Chang, Kuang-Chi Tung, Francois Guillemot, Jane E. Johnson

There was an error in the supplementary data of Development **141**, 2803-2812.

In Table S2, on the Ind.replicates tab, some values in column J (Ptf1a-het, sample1_FPKM) were misordered and hence did not correspond correctly with column A (gene); on the master tab, some rows had inadvertently been duplicated. The correct version of Table S2 now appears online.

The authors apologise to readers for this mistake.

